# The Impact of a Topical Oxygen-Releasing Gel (blue^®^m) on Deep Periodontal Pockets: A Case Report

**DOI:** 10.3390/medicina60091527

**Published:** 2024-09-19

**Authors:** Haya Alayadi, Arwa Talakey, Hajer Aldulaijan, Marwa Y. Shaheen

**Affiliations:** 1 Dental Health Department, College of Applied Medical Sciences, King Saud University, Riyadh 11433, Saudi Arabia; 2 Department of Periodontics and Community Dentistry, College of Dentistry, King Saud University, Riyadh 11433, Saudi Arabia; aatalakey@ksu.edu.sa (A.T.); haldulaijan@ksu.edu.sa (H.A.); mashaheen@ksu.edu.sa (M.Y.S.)

**Keywords:** periodontitis, periodontal pocket depth, topical oxygen therapy, gingival health

## Abstract

Periodontitis represents a prevalent oral pathological condition. Various supplementary local therapies are utilized in clinical practice. Recently introduced, topical oxygen therapy exhibits the potential to effectively hinder the growth of plaque (bacterial biofilm). Delivered in the form of an oral gel, this formulation contains cellulose, glycerol, and sodium peroxoborate, releasing topical oxygen in a regulated manner. Additionally, it discharges topical oxygen and lactoferrin in a controlled manner, with the former showcasing antibacterial properties and the latter stimulating bone cell activity. The primary objective of this particular case study is to present a scenario of periodontitis featuring deep periodontal pockets, which was effectively managed through clinical treatment with the use of adjunctive topical oxygen-releasing gel (blue^®^m).

## 1. Introduction

Periodontitis is a chronic inflammatory disease characterized by destruction of the periodontium, including bleeding on probing, deep periodontal pockets, and loss of alveolar bone, ultimately leading to tooth loss [1]. According to the 2019 Global Burden of Disease study, severe periodontitis is highly prevalent globally, accounting for 1.1 billion cases [2]. Periodontitis can negatively impact individual physical and psychological wellbeing and subsequently impairs the quality of life [1].

Microbial biofilm accumulation at or below the gingival margin is a major cause for periodontium destruction, which can ultimately progress to tooth loss. Anaerobic bacteria accumulation in deep periodontal pockets activates the host inflammatory response, leading to an environment of low oxygen levels [3,4]. Low oxygen levels in cells affect them in different ways including cell growth, proliferation, pH level, and metabolism. Additionally, low oxygen levels induce local defense reactions in inflamed tissue, which can lead to disease progression [5]. Many pathogens contribute to the inflammatory process of periodontitis including *Porphyromonas gingivalis* (*P. gingivalis*), which is one of the most prominent bacteria responsible for the initiation and progression of periodontitis [6]. As *P. gingivalis* is an anaerobic bacterium, a low oxygen environment in a deep periodontal pocket provides an ideal environment for its proliferation, which increases oxidative stress, interferes with protective mechanisms, and increases reactive oxygen species [6,7].

Non-surgical periodontal therapy alone has been widely studied and has shown to be effective in reducing pocket depths, improving clinical attachment levels, and decreasing inflammation in patients with periodontitis [2,3]. However, its effectiveness can be limited in cases of deep pockets or advanced disease [8]. Several studies have assessed the role of oxygen in wound healing, finding that increasing oxygen levels induces granulation tissue formation and collagen synthesis [8,9,10,11]. Moreover, oxygen applications have been found to stimulate angiogenesis and inhibit inflammation and infection [12]. Oxygen therapy can be administered either systemically or topically. Topical oxygen therapy releases controlled active oxygen molecules and is available in different forms including toothpaste, mouth wash, mouth foam, and oral gel formulations. Studies on topical oxygen therapy have found that its effects mimic the effect of subgingival scaling in terms of plaque control and periodontal tissue inflammation [13,14]. Some studies suggest it may have a positive effect on gingival inflammation and pocket depth reduction, but results are not as consistent or substantial as those of traditional therapy [9,10,11,12,13]. The combination of non-surgical periodontal therapy and topical oxygen therapy has shown the most promising results in recent studies [12,13,14]. This approach appears to leverage the benefits of mechanical debridement with the potential antibacterial and healing-promoting effects of oxygen therapy. The aim of this study is to report a case of periodontitis with deep periodontal pockets, which was successfully treated clinically with topical oxygen-releasing gel (blue^®^m).

## 2. Case Presentation

A single 36-year-old woman presented herself for examination at King Saud University in the dental clinic in the Department of Periodontics and Community Dentistry, with the chief aim of cleaning her teeth. The lady had received a thyroidectomy three months prior to her dental appointment and, since then, had been taking 100 mg of Thyroxine. Besides that, she had no other underlying medical conditions.

**Figure 1:**
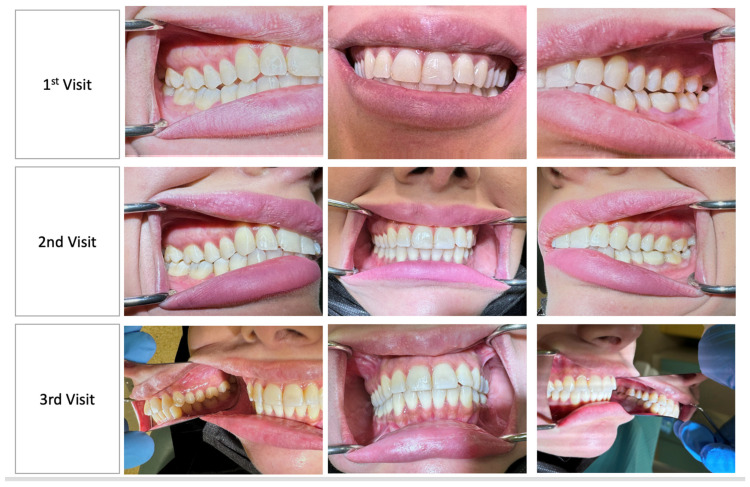
Intraoral examination at first visit: baseline; second visit: + 7 days from baseline; and 3rd visit: +14 days from baseline.

The reason for the patient’s dental care was a regular dental visit, as she attended the dental clinic with no pain or need for urgent emergency care, yet her most recent dental appointment was 1 year prior. Her oral hygiene routine included the use of a soft electronic toothbrush used once a day and water floss used once daily. A visual analogue scale confirmed her asymptomatic status by showing that the patient presented to the dental appointment with no pain (Figure 1).

**Figure 2:**
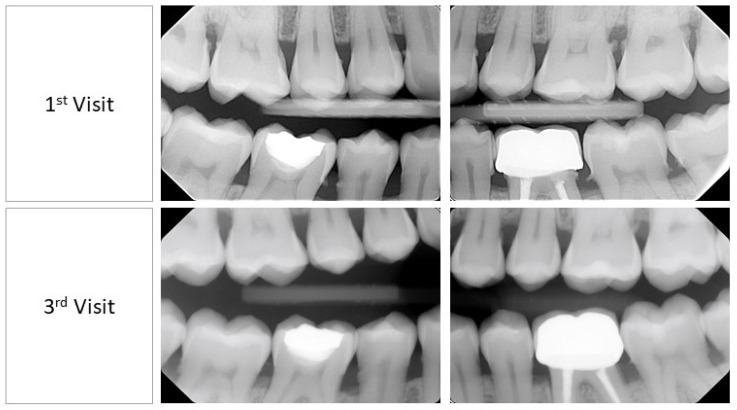
Bitewing radiographic examination at first visit: baseline and third visit: +14 days.

**Figure 3:**
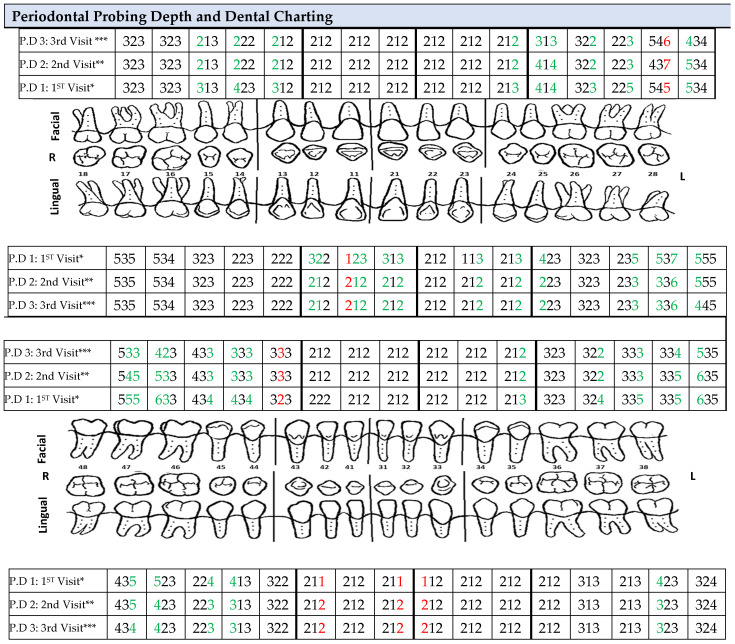
* P. D 1: first visit: periodontal probing depth at baseline; ** P.D 2: second visit: periodontal probing depth at +7 days from baseline; and *** P.D 3: third visit: periodontal probing depth +14 days from baseline.

A clinical examination revealed interdental papilla and marginal gingival erythema (color), soft and spongy consistency, loss of stippling texture, and a generalized rolled gingival margin (Figure 1). This provided us with a wide view of the patient’s clinical gingival health including papilla, gingival margin, and gingival consistency. An in-depth view presented using a bitewing radiographic examination revealed generalized bone loss as a result of papillary contours (Figure 2). The dental indices utilized in this case study were the gingival index by Loe and Silness [15], plaque index by O’Leary et al. [16], and bleeding on probing (BOP) by Ainamo and Bay [17]. The examination at first appointment revealed the following: gingival index: 2; plaque index: 21%; and bleeding index: 14%. Hence, dental charting was utilized for a detailed illustration of each site’s conditions, which uncovered deep pockets in multiple sites (Figure 3).

The suggested plan of care included having the patient consent to participate in this case study and to take pictures of the case, as well as share her X-rays for research purposes. The plan of care additionally included scaling and root planing (SRP) for deep pockets assessed through periodontal pocket depth (PPD), prophylaxes and fluoride application, and the use of topical oxygen-releasing gel blue^®^m [18] applied twice per day by the patient to the areas of the deep pockets. The plan of care also included patient motivation through oral hygiene instructions—1/use of a soft toothbrush twice per day, 2/modified bass technique, and 3/use of waxed dental floss once per day. The proposed follow-up was +7 days and +14 days from the first visit in order to assess periodontal pocket depth healing after the application of topical oxygen-releasing gel blue^®^m.

Dental indices after 7 days from baseline revealed the following: gingival index: 2; plaque index: 50%; and bleeding index: 38%. On the other hand, after 14 days, the dental indices showed gingival index: 2; plaque index: 15%; and bleeding index: 9%. Intraoral conditions on the second and third appointments are shown in Figure 1 and Figure 2. It is worth noting that the visual analogue scale on the third appointment (after 14 days) reported no differences than what was reported in the first visit.

Periodontal pocket depth (PPD) measurements and dental charting revealed reduced PPD (1–2 mm total reductions) in follow-up appointments (+7 and +14 days from baseline), as illustrated in Figure 3. This Figure details periodontal probing depth at baseline, after 7 days, and after 14 days from baseline. It also reveals gradual improvement in periodontal pocket depth marked in green for the following teeth:

- Facial side: 16, 15, 14, 23, 24, 25, 26, 28, 33, 35, 36, 37, 38, 46, 47, 48;
- Lingual side: 13, 12, 11, 22, 23, 24, 26, 27, 28, 37, 46, 48.

Conversely, some of the following sites showed increased periodontal pocket depth marked in red:

- Facial side: 27 and 45;
- Lingual side: 12, 31, 42, 43.

Additionally, areas marked in black indicate sites that showed no change over the course of the treatment period.

We propose that after the initial removal of calculus, some areas of the gingiva might have remained inflamed and swollen [19] or there might have been residual subgingival calculus [20]. This condition could have impeded the dentist’s ability to fully access the base of the periodontal pocket during the initial examination. As the gingival tissue began to heal and recover following the treatment, it allowed for a more accurate assessment of the true pocket depth. Consequently, during the final follow-up appointment, these particular sites showed a reduction in pocket depth, reflecting the progression of the healing process and the effectiveness of the treatment.

## 3. Discussion

This case report presents a unique and valuable contribution to the field of periodontal treatment, particularly for patients with complex medical histories. It showcases a novel approach using a recently introduced topical oxygen-releasing gel (blue^®^m) as an adjunctive therapy to traditional periodontal treatment, a method that is currently understudied. This case was further complicated by the patient’s recent thyroidectomy, which adds an intriguing layer to the treatment outcomes. Notably, the case demonstrated significant improvement of periodontal pocket depth within a remarkably short period of 14 days, despite the patient’s medical history and the severity of the initial condition. The effectiveness of combining mechanical debridement with the application of the topical oxygen-releasing gel highlights a potentially synergistic approach to periodontal treatment. Given the limited existing literature on this particular formulation, this comprehensive case report, complete with detailed documentation of dental indices, periodontal pocket depths, and visual evidence, provides valuable insights into its efficacy. The inclusion of the patient’s perspective on the treatment adds a unique element to this report. Ultimately, the success observed in this case suggests the potential for developing new clinical guidelines that incorporate topical oral oxygen therapy in the treatment of gingivitis and periodontitis, making it a significant contribution to the evolving landscape of periodontal care.

The formulation of topical oxygen therapy (blue^®^m) was recently created by a research team under the leadership of Dr. Peter Blijdorp. The uniqueness of this formulation suggests potential in impeding the growth of plaque (bacterial biofilm) in clinical settings [18]. This was a discreet development; therefore, there exists a limited body of literature that demonstrates the efficacy of a topical oxygen therapy formulation (blue^®^m). Hence, the primary objective of this case study is to present a successful clinical intervention for a periodontitis case with deep periodontal pockets, utilizing a topical oxygen-releasing gel (blue^®^m). It is important to highlight that the patient had undergone a thyroidectomy in the preceding three months, which could potentially be linked to oral manifestations, particularly in terms of compromised periodontal health and delayed soft tissue healing [21].

The patient’s treatment outcome showed improved color—pale pink—a firm consistency, stippled texture, and scalloping with sharp-edged margins, all of which are a characteristic of healthy gingiva [22]. In terms of indices, the plaque and bleeding indexes were observed to have an overall reduction of 6% and 12%, respectively, from the baseline to the final follow-up appointment. The primary outcome in this instance was the presence of deep pockets, which exhibited an improvement in most areas with a total decrease of 1–2 mm within a span of 14 days. Given the oral manifestations following a thyroidectomy, the expected outcome included delayed healing and poor periodontal health. Nonetheless, some sites did experience an increase in pocket depth during the second visit. Our fundamental hypothesis is that following calculus removal, the gingiva remained swollen and inflamed in certain areas [19], or there was a residual subgingival calculus [20], therefore hindering the clinician’s access to the base of the periodontal pocket during the baseline appointment. This allowed the gingival tissue to regenerate and recuperate during the post treatment evaluation at the baseline appointment, revealing the actual pocket depth. Consequently, these specific areas exhibited a decrease in pocket depth during the final follow-up examination on the third visit.

Moreover, it is noteworthy to highlight that this effective approach involved the utilization of mechanical debridement such as scaling and root planing alongside the application of a topical oxygen-releasing gel (blue^®^m). This integrated method has proven to be efficacious. It is crucial to acknowledge that comparable outcomes cannot be achieved without standard clinical interventions.

The case being presented illustrates the successful clinical management of a periodontitis case characterized by deep periodontal pockets via application of topical oxygen-releasing gel (blue^®^m). A similar outcome was observed in case reports by Basudan [12] and her colleagues, wherein the utilization of topical oxygen via toothpaste and gel for a period exceeding 14 days led to the resolution of all clinical manifestations of gingivitis and the absence of bleeding upon probing in a gingivitis case. Furthermore, the publication documented an instance of periodontal disease exhibiting enhanced periodontal probing depth after a duration of two months of topical oxygen application through toothpaste and gel. This particular case displayed no bleeding upon probing, with the periodontal probing depth limited to a maximum of 3 mm [12].

## 4. Conclusions

To conclude, the application of topical oral oxygen therapy (blue^®^m) twice daily serves as an effective treatment and valuable complement to mechanical debridement and scaling and root planing in the management of deep periodontal pockets. Additionally, topical oral oxygen therapy (blue^®^m) proved its effectiveness, regardless of the patient’s complicated medical history, which may have had a negative impact on the healing process. According to the patient’s perspective on the ease of use of the gel, it might be a valuable supplement to home care regimens, possibly improving treatment compliance. Nonetheless, additional extensive, extended, comparative research is essential to establish clinical guidelines founded on topical oral oxygen therapy.

## 5. Patient Prospective

I would consider blue^®^m mouth gel to be effective due to the sense of relief it provided after application, its ease of use, and its ability to reduce gum swelling.

## Data Availability

All data are contained within the article.
